# Prediction and assessment of xenoestrogens mixture effects using the *in vitro* ERα-CALUX assay

**DOI:** 10.3389/ftox.2023.1252847

**Published:** 2023-12-08

**Authors:** Marc Elskens, Imke Boonen, Steven Eisenreich

**Affiliations:** ^1^ Laboratory for Analytical and Environmental Chemistry, Chemistry Department, Vrije Universiteit Brussel, Brussels, Belgium; ^2^ Hydrology and Hydraulic Engineering Department, Vrije Universiteit Brussel, Brussels, Belgium

**Keywords:** xenoestrogens, *in vitro* assays, mixture effects, food contact materials, independent action, concentration addition

## Abstract

**Introduction:** Many natural or synthetic compounds used in foods, dietary supplements, and food contact materials (FCMs) are suspected endocrine disruptors (EDs). Currently, scientific evidence to predict the impacts on biological systems of ED mixtures is lacking. In this study, three classes of substances were considered: i) phytoestrogens, ii) plant protection products (PPP) and iii) substances related to FCMs. Fourteen compounds were selected based on their potential endocrine activity and their presence in food and FCMs.

**Methods:** These compounds were evaluated using an *in vitro* gene expression assay, the ERα-CALUX, to characterize their responses on the estrogen receptor alpha. Cells were exposed to fixed ratio mixtures and non-equipotent mixtures of full and partial agonists. The concentration-response curves measured for the three classes of compounds were characterized by variable geometric parameters in terms of maximum response (efficacy), sensitivity (slope) and potency (median effective concentration EC50). To account for these variations, a generic response addition (GRA) model was derived from mass action kinetics.

**Results:** Although GRA does not allow us to clearly separate the concentration addition (CA) and independent action (IA) models, it was possible to determine in a statistically robust way whether the combined action of the chemicals in the mixture acted by interaction (synergy and antagonism) or by additive behavior. This distinction is crucial for assessing the risks associated with exposure to xenoestrogens. A benchmark dose approach was used to compare the response of phytoestrogen blends in the presence and absence of the hormone estradiol (E2). At the same time, 12 mixtures of 2–5 constituents including phytoestrogens, phthalates and PPPs in proportions close to those found in food products were tested. In 95% of cases, the response pattern observed showed a joint and independent effect of the chemicals on ER.

**Discussion:** Overall, these results validate a risk assessment approach based on an additive effects model modulated by intrinsic toxicity factors. Here, the CA and IA approaches cannot be distinguished solely based on the shape of the concentration response curves. However, the optimized GRA model is more robust than CA when the efficacy, potency, and sensitivity of individual chemical agonists show large variations.

## 1 Introduction

Endocrine disruptors (EDs) are substances that affect the endocrine system and have adverse effects on the health of an organism and/or its offspring. EDs affect the hormonal balance and impact human health by modifying functions such as growth, development, sexual and reproductive function, behavior, obesity, and blood circulation (Diamanti-Kandarakis et al., 2009; Guarnotta et al., 2022). The increasing incidence of endocrine diseases and associated disabilities is assumed related to the increasing exposure of the population to EDs (Ahn and Jeung, 2023). These chemicals disrupt the body at different levels, often in discrete ways and in doses as low as the order of physiological hormone concentrations (Marlatt et al., 2022). The impact on human health is even more worrying since the effect of these substances on fetal development may be irreversible. It was estimated that the annual cost of EDs for European society is of the order of hundreds of billions of euros per year (Trasande et al., 2016).

According to REACH (updated list in April 2022), 24 chemicals were identified and legally adopted as endocrine disruptors for humans. These include phthalates, and the bisphenol and phenol groups. However, many other compounds are considered potential EDs including antioxidants, pesticides, biocides, detergents, metals, plasticizers, and organometallic compounds (Mukherjee et al., 2021; Wiesinger et al., 2021; Imparato et al., 2022). Diet is an important exposure route for these known or potential EDs, which enter the food chain in different ways. They can occur naturally as phytoestrogens, deliberately added as additives, or can contaminate food through production, processing, transportation, and migration from food contact materials (FCM) (Ong et al., 2020; Wiesinger et al., 2021). One of the major challenges with current testing and regulation of EDs is that they are assessed on an individual basis, whereas in real life the population is exposed to multiple compounds (“mixtures”) simultaneously. EDs enter the human body as complex mixtures, usually through chronic low-dose exposure. This implies that the organism may be exposed to the same compound from several sources or that a consumer product may contain several known or potential EDs (Kumar et al., 2020; Darbre, 2022).

Current practice is to determine the toxicity of individual compounds, after which the mixture is considered “tolerable” when the toxicity or concentration of individual compounds is below experimentally defined toxicity threshold values (Heys et al., 2016). However, the fact that the toxicity of a single compound is known does not guarantee its behavior when combined with other substances. Compounds that share a common mechanism of action may be able to cooperate with each other and induce a measurable effect even if they are individually present below their No Observed Adverse Effect Level—NOAEL (Silva et al., 2002). These joint effects therefore complicate risk assessment, hence the need to extend current methods of toxicological assessment (Perkins et al., 2019).

The two most used models to predict joint effects in mixtures are concentration addition (CA) and independent action (IA), both having various names in the literature (Cedergreen et al., 2008; Medlock Kakaley et al., 2019; Hsieh et al., 2021). The theoretical principle of CA is that the chemicals in the mixture share the same target molecule (same mode of action) and dose-response curves differ only in potency (Gottschalk and Dunn, 2005). They can, therefore, be considered as dilutions of each other. In the IA model, the chemicals in the mixture are assumed to have different modes of action, thus affecting different potential biological target sites. Since they act independently, the overall mixture might show no response when the compounds are present below their NOAEL (Martin et al., 2009). A common assumption between CA and IA is that chemicals in a mixture do not interact physically, chemically, or biologically. However, interactions between different chemicals are possible and can have a substantial impact on the overall potency or toxicity of the mixture. Either the result of the mixture exceeds that predicted by the sum of the individual effects, which is called synergy, or the result of the mixture is less than that expected, which is called antagonism (Cedergreen et al., 2017; Martin et al., 2021). A thorough study of the literature shows that cocktail effects for EDs with interactions are only observed in 11% of the cases studied. Overall, the deviations were small, and very often additivity could be used as the default concept to predict mixture outcomes (Martin et al., 2021).

In this study, to better appreciate these possible cocktail effects, the estrogenic activity of mixtures consisting of several known and/or potential EDs related to food and FCM was studied using the bio- ERα-CALUX assay. The experimental results were compared to the activity predicted by models derived from CA and IA concepts, to test whether the mixtures followed the additivity principle or showed signs of interaction. Mixtures have also been prepared containing compounds below their activity threshold to determine if the overall mixture activity was significant, illustrating the so-called “something out of nothing” paradigm (Silva et al., 2002).

## 2 Material and methods

### 2.1 Prioritization of potential estrogenic disruptors

Potential endocrine active substances present in foods for study come from the categories: i) phytoestrogens, ii) biocides and phytosanitary products and iii) substances present in the form of additives or present in FCMs. For each of these categories, a list based on the presence on the Belgium food market was established (see Bel et al., 2016), and a selection based on the potential for endocrine disruption was made after literature review (ex. Brouwers et al., 2009; EFSA, 2019). The selection of these compounds of interest guided the selection of foods and FCMs, which were analyzed by LC/GC-MS/MS as part of the FPS Public Health project ENDFOODTOX (RF 18/6326).

### 2.2 Analytical standards

The following analytical standards were purchased: i) from Sigma Aldrich: butylated hydroxytoluene (BHT), Benzophenone, Bis (2-ethylhexyl) phthalate (DEHP), Dibutyl phthalate (DBP), Diisobutyl phthalate (DIBP), Kaempferol, and Equol, ii) from Phytilab: Dadzein, Genistein, 6-Prenylnaringenin (6-PN), 8-Prenylnaringenin (8-PN) and Enterolactone and iii) from LGC: Benzyl butyl phthalate (BBP) and Triadimenol.

Each reference material was solubilized in appropriate solvents to make stock solutions at a concentration of 1 mg/mL. Most compounds were solubilized in MeOH, except daidzin and genistin, which required DMSO. BHT is soluble in MeOH, but anhydrous ethanol or hexane is required to prevent its degradation. Working standards at 20 μg/mL and 10 μg/mL were prepared in MeOH and ACN. Stock and working standards were stored at −20°C and retained for a maximum of 6 months.

### 2.3 Assessment of endocrine activities using the ERα-CALUX assay

The recombinant human breast cancer cell line VM7Luc4E2 (variant MCF7, formerly known as BG1Luc4E2) was used to determine estrogenic activities. These cells express ERα endogenously but lack any functional ERβ (Rogers and Denison, 2000; Brennan et al., 2016). The bioanalytical procedure and data analysis are described in (Elskens et al., 2023).

### 2.4 Interpretation of concentration-response curves for mixtures

A growing body of experimental evidence indicates that the *in vitro* activities of chemical mixtures can be predicted from the overall response of their individual components using the concept of the concentration addition model-CA (Evans et al., 2012). Mathematically, CA models assume that all dose-response curves are characterized by similar threshold values and similar efficacy and that their slope-values are not significantly different from 1.

$${EC}_{X_{MIX}}={\left(\sum_{1=1}^{n}\frac{f_{i}}{{EC}_{X_{i}}}\right)}^{-1}$$ (1)

where ${EC}_{X_{i}}$ is the effective concentration of compound *i* alone causing an effect ***X***, ***f***_***i***_ is the fraction of ***i*** present in the mixture and ${EC}_{X_{MIX}}$ is the effective concentration of the mixture causing the same effect ***X***. To apply Eq. 1, the individual potencies of the compounds present in the mixture are required (Supplementary Table S1).

Assumptions that all concentration-response curves are characterized by similar threshold, efficacy and slope-values are not valid for phytoestrogen response on ER (Supplementary Table S1). The Hill slope are frequently different from 1, and the maximum response fluctuate depending on the presence of full-, partial-, or supra-agonists. To correct for these inconsistencies, a logistic function was derived from the mass action kinetics using chemical equilibrium reactions:

$$R+\sum_{i=1}^{n}n_{i}∙L_{i}\Leftrightarrow \sum_{i=1}^{n}{RL}_{n_{i}}$$ (2)

where ***R*** is the receptor protein concentration, ***L***_***i***_ the free unbound ligand concentration with ***n***_***i***_ binding sites. From the law of mass action, the apparent dissociation constant ***K***_***i***_ for ligand ***L***_***i***_ is given by:

$$K_{i}=\frac{\left[R\right]∙{\left[L_{i}\right]}^{n_{i}}}{\left[RL_{in_{i}}\right]}$$ (3)

The ratio of occupied receptor to total receptor is then:

$$\Phi =\frac{\sum_{i=1}^{n}{RL}_{in_{i}}}{R+\sum_{i=1}^{n}{RL}_{in_{i}}}$$ (4)

Solving for the receptor activity, one finally obtains:

$$y_{i}=y_{0}+\left(m-y_{0}\right)∙\frac{\sum_{i=1}^{n}a_{i}∙{\left(\frac{x_{i}}{c_{i}}\right)}^{n_{i}}}{1+\sum_{i=1}^{n}{\left(\frac{x_{i}}{c_{i}}\right)}^{n_{i}}}$$ (5)

which is a generic formulation of the Hill equation for several binding ligands. In Eq. 5 ***y***_***0***_ and ***m*** are the lower and upper asymptote for E2, ***a***_***i***_ = efficacy, ***c***_***i***_ = EC_50_, ***n***_***i***_ = Hill slope of ligand ***L***_***i***_.

The parameter ***n***_***i***_ represents a “sensitivity coefficient” describing the slope of the tangent to the dose response curve at its inflection point for each ligand. Eq. 5, referred to as GRA, is hereafter considered equivalent to a mixed CA/IA approach, because it makes possible to consider the variability of the parameters defining the potency, sensitivity, and amplitude of the concentration-response curves for each compound tested in a mixture. Values for ***a***_***i***_, ***c***_***i***_, and ***n***_***i***_ are summarized in (Supplementary Table S1). CA and IA models assumes the additivity principle between tested compounds. It is the default assumption for predicting and evaluating the mixing effect. The accuracy of these models was assessed by comparing the predicted curves against the observed values with the root mean square error (RMSE), which measures the average difference between a model’s predicted values and the actual values. In addition, the measured concentration-response curves of the mixture are compared with those obtained by modelling, and the equivalence hypothesis rejected if the measured curve is outside the 95% confidence interval of the theoretical models obtained by means of Monte-Carlo simulations carried out using XLSTAT-Sim (XLSTAT version 2023.1.5) according to normal and/or uniform distribution laws.

### 2.5 Quality control and quality assurance (QC/QA)

The best-fit parameters for the concentration response-curves were obtained by minimizing the least squares residuals using a Levenberg-Marquardt algorithm in R version 4.1.1-2021-08-10 (Elskens et al., 2011). For the standard E2 curves, the parameter-values (95% CI) under repeatability conditions and the goodness of fit criteria (min—max) are given in Supplementary Table S1. Sample activity below the threshold values of 20% was designated as < LoQ. Sample activity below the threshold values of 10% was designated as not detectable (<LoD). Quality control samples were systematically performed in triplicate on the 96-well plates. They consisted of standard at the half maximal effective concentration (EC_50_). The recovery rates were between 89% and 120% for E2, results which can be considered as satisfactory.

## 3 Results and discussion

### 3.1 Rationale and assumption behind the derivation of the Ca and GRA models

Concentration-response curves were used to study the *in vitro* effects of mixtures, assuming that the bioactivity of these mixtures followed by default the principle of additivity according to Eq. 1. This model assumes that all the ligands act in the same way as dilutions of each other. While the binding affinity on the receptor may differ between compounds (i.e., relative potency), they all exhibit the same slope (i.e., Hill coefficient) and maximum response (i.e., efficacy), resulting in parallelism between sets of concentration response curves (Gottschalk and Dunn, 2005). However, for the chemicals investigated in this study, the parallelism hypothesis is not valid; the efficacy (***a***) varying between 0.5 and 2 and Hill coefficient (***n***) between 0.5 and 5 (Supplementary Table S1). Consequently, a generalisation of the Hill-Langmuir function (Eq. 5) was derived from the mass action kinetics (Eq. 2) assuming that each ligand is characterised by an intrinsic potency (***c***_***i***_), sensitivity (***n***_***i***_) and maximum response (***a***_***i***_), providing a generic response addition model (GRA).

**FIGURE 1:**
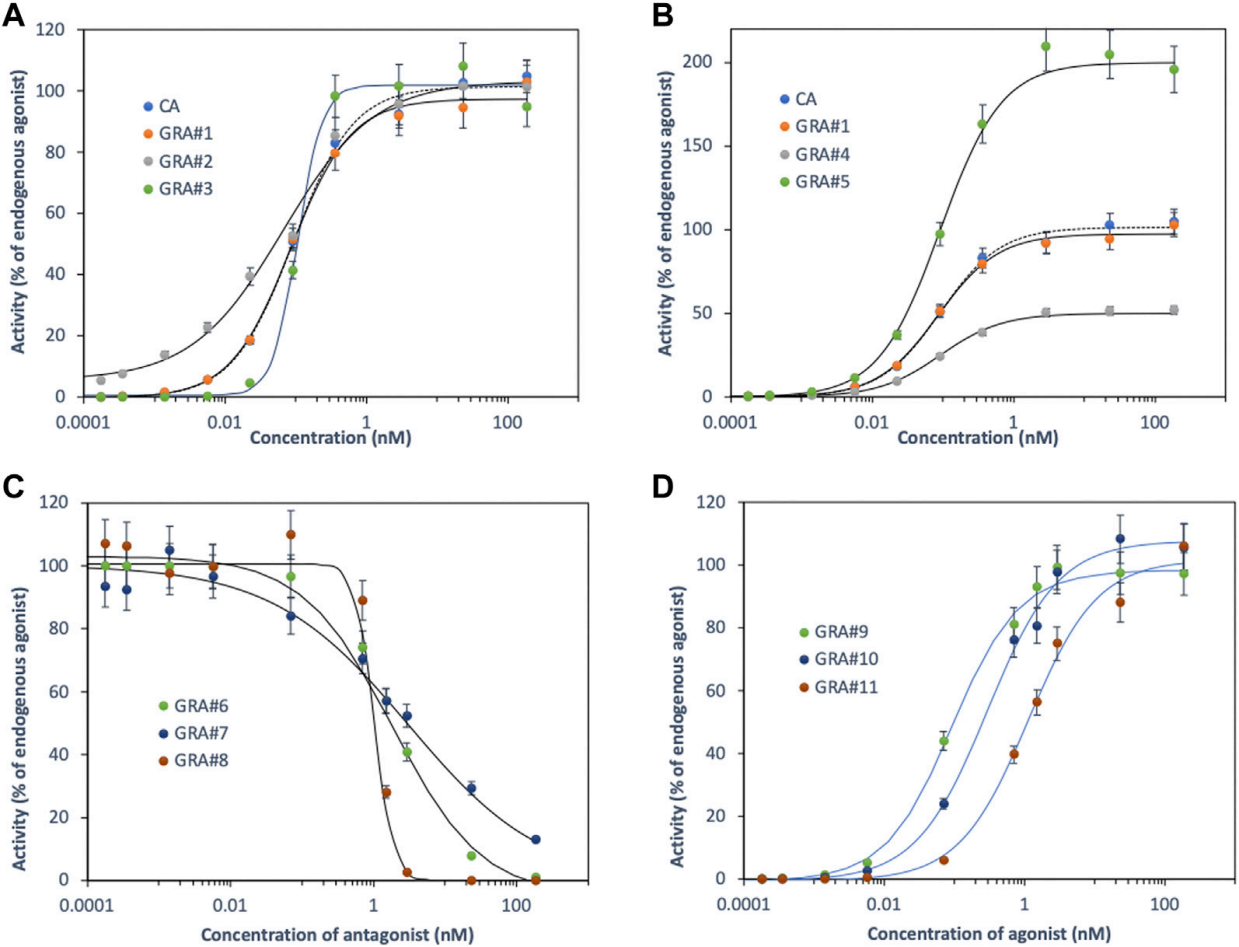
Concentration response-curves generated by CA and GRA for equipotent binary mixtures of fictitious agonists/antagonists (see Supplementary Table S2).

Binding curves showing the characteristically sigmoid generated by CA and GRA for an equipotent binary mixture of fictitious agonists are illustrated in Figure 1. The effect of random noise (7%) on the shape of the response curve and the empirical adjustment of parameters was investigated (Supplementary Table S2). Both models give non-statistically different values for the fitted parameters when compared to actual values at the 5% level of significance. CA is a special case of GRA for (***a***_***i***_ = 1, ***n***_***i***_ = 1) but as ***n***_***i***_ or ***a***_***i***_ increases, the saturation curves become steeper or higher for GRA (Figures 1A, B). The question arises as to the physiological significance of these parameters in the CALUX assay. The Hill coefficient plays a leading role in the study of ligand-receptor interactions, measuring the number of binding sites in cooperating systems (Abeliovich, 2005). For ***n***_***i***_ >1, when a ligand is bound to the receptor, its affinity for other ligands increases. For ***n***_***i***_ <1, once a ligand is bound to the receptor, its affinity for other ligands decreases. Finally, for ***n***_***i***_ = 1, the affinity of the receptor for a ligand does not depend on whether other ligands are already bound to the receptor. In practice, however, ***n***_***i***_ in the CALUX assay is an ‘apparent coefficient’ reflecting a series of processes. After ligand binding to the cytosolic receptor, translocation of the newly formed dimeric complex in the nucleus and binding to DNA, the CALUX cells induce different protein activities via transcription, translation, and enzymatic expression. While we cannot rule out positive or negative cooperative binding for ***n***_***i***_ ≠1, neither can we rule out indirect or cumulative effects on either of the reaction sequences. The same reasoning applies to ***a***_***i***_. The concept of “super agonism” was described in the early 1980 s, in relation to peptide hormone analogues that produced greater functional responses than endogenous agonists. Whether these compounds can actually be more effective than endogenous agonists have long been debated, but relatively recent pharmacological evidence has indicated that super agonists may be more than an artefact (Langmead and Christopoulos, 2013). Consequently, it is risky to assume that the functional affinity operating at the level of the whole cell reflects the molecular ligand-receptor binding affinity; care must be taken not to over-interpret the significance or reliability of the fitted parameters, even if it can be shown empirically that ***a***_***i***_ and ***n***_***i***_ are statistically different from 1. Moreover, the GRA model allows the effect of antagonism to be considered. Whether ***a***_***i***_ of a given compound tends towards 0, it can bind to the receptor but does not activate it (Figure 1C). This results in a decrease in agonist activity in the presence of increasing concentrations of antagonist, the inhibitory effect of which being estimated by the IC_50_ (concentration of antagonist inhibiting 50% of the agonist response). Supplementary Table S2 shows how the parameters fitted with a background of 7% compare with the actual model outputs. As previously, the shape of the concentration-response curves is modulated by ***n***_***i***_. The inhibition reflects a reversible competitive antagonism. The latter can be overcome. At high concentrations, the effect of the antagonist is no longer observed but the sigmoid agonist curve shifts to the right and its “apparent” potency is, therefore, reduced (EC_50_ increases). The higher the antagonist concentration, the greater the displacement (Figure 1D).

GRA, therefore, appears to be a generalisation of CA and, like it, assumes an additive pattern. The concentration-response curves being modulated by the intrinsic ***a***_***i***_ and ***n***_***i***_ values, allow us to consider both mixtures of competing agonists and agonists/antagonists. However, this approach does not allow us to assess the synergy between endogenous and exogenous agonists, i.e., the sigmoid curve of the mixture shifts to the left and its “apparent potency” increases (EC_50_ decreases). Such synergies have been less frequently reported and might occur at lower concentration ratios (Cox et al., 2021). Interaction is, therefore, expressed as changes in amplitude (increase/decrease in efficacy), potency (increase/decrease in binding affinity to the receptor), sensitivity (increase/decrease in slope response) or all three together (Indurthi and Auerbach, 2021). It is essential to assess these interactions, for example, when antagonists or toxic compounds are present in a sample, the CALUX response may be partially masked or amplified. The aim is to determine i) which CA or GRA approaches give the best description in mixture of real life food samples, and ii) whether the investigated chemicals follow an additive pattern or show signs of synergistic and/or antagonistic activity. A random noise of 7% on the CALUX experimental data is close to reality for triplicate measurements. This results in RMSE values between 2.7 and 4.9 and RSQ values greater than 0.9903 (Supplementary Table S2). These goodness-of-fit parameters summarise the difference between the observed and predicted values that can be expected if the CA or GRA models describe the set of observations satisfactorily.

### 3.2 Mixture effects of phytoestrogens at EC_10_ and EC_50_ in the presence and absence of E2

In these experiments, a mixture of 4 phytoestrogens (8-PN, daidzein, kaempferol and equol) was studied using the benchmark dose (BMD) approach. The concentration-response curves measured at low dose (EC_10_) and at their half-maximal responses (EC_50_) were compared with those predicted by the CA and GRA models (Eqs 1, 5), respectively). All measured activities were expressed as % relative to the maximum response of the reference ligand E2; the negative control is represented by the DMSO blank.

**FIGURE 2:**
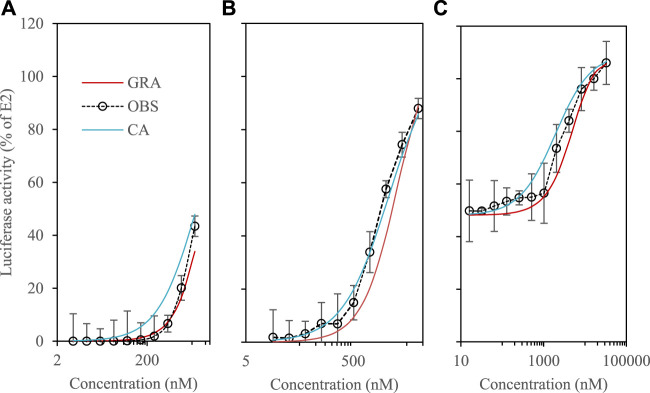
Luciferase activity in VM7Luc4E cells for phytoestrogens. #a: 8-PN, daidzein, kaempferol and equol at EC_10_; #b: 8-PN, daidzein, kaempferol and equol at EC_50_; #c: E2 at EC_50_ + Mix daidzein, kaempferol and equol (see Supplementary Table S3).

Figure 2A depicts that joint effects were detected at EC_10_ and there were no statistical differences between the GRA-predicted and observed curves. The two curves overlap, and the observations are within the interval defined by the two models. RSQ and RMSE values are reported in Supplementary Table S3. At EC_50_, the observed curve is slightly shifted to the left and the fit is better with the CA model (Figure 2B). However, the observations are most always included in the interval defined by GRA and CA.

We then checked whether the presence of phytoestrogens could modify the response of E2 tested at its EC_50_ (Figure 2C). At high dilutions of the phytoestrogen mixture (≤0.1 μM), the E2 response was not significantly modified. At higher concentrations (≥1 µM), the presence of phytoestrogens strengthens the E2 agonist response. Again, the observations are included in the interval defined by GRA and CA, and the RSQ & RMSE-values of both models are comparable (Supplementary Table S3). The mixture does not strongly modify the activity of E2. At high dilutions, there is a slightly higher overall activity of the mixture compared to the predictions, which decreases approaching saturation (Figure 2C). The fact that concentration-response curves can be steeper at lower exposure levels has been described for a variety of environmental toxicants, including lead, arsenic, and benzene (Hornung and Lanphear, 2014). Overall, current research on synergistic mixing effects is limited, and when discussed in the literature, their occurrence is low. A study on pesticide mixtures, for example, which examined the effects of mixing in realistic low-dose mixtures, reported that only 5% of the mixture combinations tested exhibited synergistic interactions (Cedergreen, 2014). Here, if there is a synergistic effect, it remains weak, and the GRA model provide overall a reasonable approximation of the behavior of the mixtures.

### 3.3 Mixtures at observed concentration ratios in food samples

**FIGURE 3:**
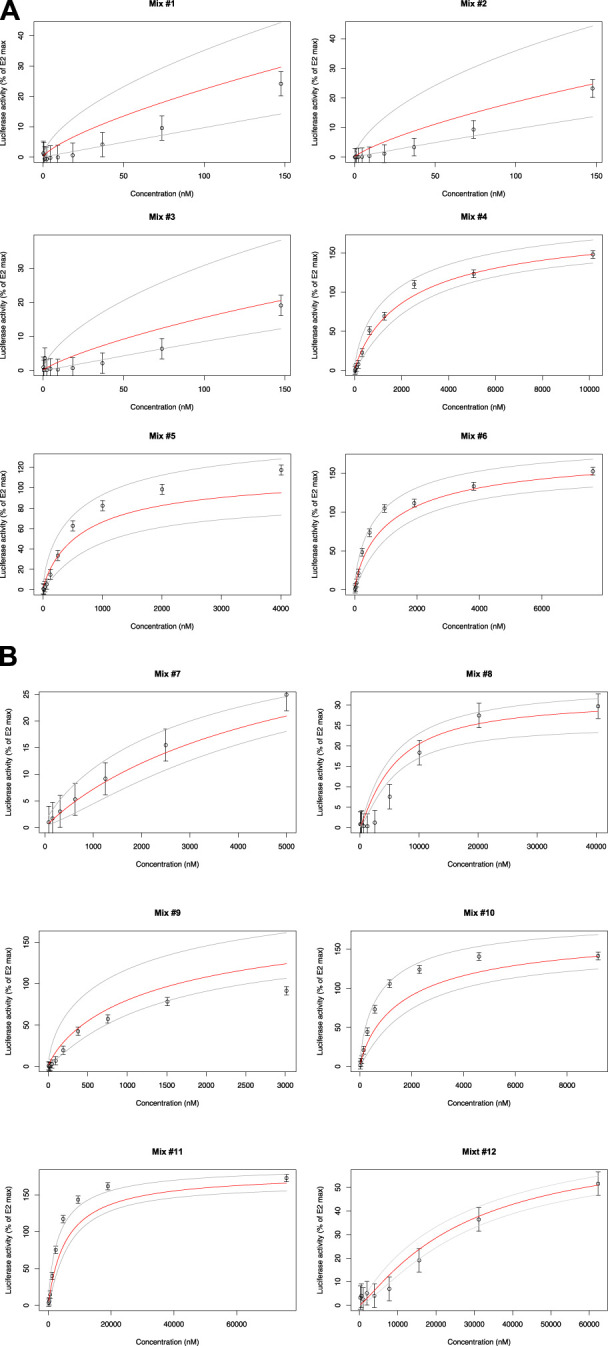
Luciferase activity in VM7Luc4E cells for Mix #1 to #6 (see Supplementary Table S3). Luciferase activity in VM7Luc4E cells for Mix #7 to #12 (see Supplementary Table S3).

Twelve mixtures of 2–5 compounds of phytoestrogens, phthalates, food additives and plant protection products were prepared according to concentration ratios found in real food samples, and their estrogenic activities were tested using ERα-CALUX. Concentration ratios were determined by LC/GC-MS/MS. as part of the ENDFOODTOX project funded by FPS Public Health (RF 18/6326). More information about how the food samples were selected can be found in the Supplementary Material section. The concentration-response curves obtained for the 12 mixtures and the corresponding model predictions are shown in Figure 3. Overall, the GRA model gave better results for these mixtures than the CA model (Supplementary Table S3). The observed experimental results are within the 95% CI of the prediction model. Consequently, no statistically significant differences between observation and prediction could be reported. There were some deviations from the expected mean activity with mixtures #1, #2, and #3 (Daidzein, Genistein) showing slightly below-average activities and mixtures #5 (Daidzein, Genistein, 6-PN, Kaempferol) and #6 (Daidzein, Genistein, 6-PN, Enterolactone) showing slightly above-average activities. Mixtures #10 (Daidzein, Genistein, 6-PN, Enterolactone, BHT) and #11 (Daidzein, Genistein, DIBP, Benzophenone, Triadimenol) show observations that fall on the leftmost edge of the 95 CI %, which could suggest the possibility of synergy in these mixtures.

However, the experimental uncertainties (particularly for #9), do not allow us to conclude that there is a significant difference amongst the observed results. Triadimenol is present only in #11, while #10 and #12 are the only ones to contain BHT. Mixture #12, however, contains a lower BHT concentration, which might suggest that the potentially increased potency compared to the expected activity could be due to the antioxidant BHT in #10 and the fungicide triadimenol in #11. Synergistic effects with fungicides have already been described (Rondeau and Raine, 2022). This has been documented for triadimenol, which exhibits synergy effects when used in combination with other triazole-based fungicides (Li et al., 2018). For BHT, no description of synergistic activity in mixtures has been reported in the literature. Overall, our results show that the additive model is a sufficiently robust default model to describe mixture effects. Similarly, Yu et al. (2019) determinized the estrogenic activity of environmental EDs using the yeast estrogen screening test (YES) which suggested additive effects best described the observed results. Furthermore, more recent work on the combined toxicity of EDs revealed that the CA approach provided reliable estimates for describing joint effects when the compounds tested shared a common mode of action (Hamid et al., 2021).

The CA model assumes, however, that all the chemicals in the mixture behave as a dilution of each other. Mathematically, this implies that the concentration-response curves for each compound follow a parallel-line logistic regression model (Gottschalk and Dunn, 2005). It also means that the Hill slope is not significantly different from 1 and that all the concentration-response curves of the compounds in the mixture have equivalent background and maximum responses (Cedergreen et al., 2008). These assumptions do not apply to our CALUX measurements, where the Hill coefficient may differ from 1 and the maximum responses may fluctuate greatly, particularly in the case of phytoestrogens (Supplementary Table S1). Daidzein and genistein, for example, are supra-inductive agonists (180%–200% efficacy), while kaempferol (60% efficacy) is a partial agonist. An additive model was therefore adapted, to simultaneously consider variations in the efficacy, potency, and sensitivity of the different compounds. Monte Carlo simulations were then used to test the robustness of the additivity hypothesis. This made it possible to reconstruct concentration response curves for highly variable experimental conditions and to accurately predict the activity of the mixtures in most cases. The GRA approach therefore appears to be a suitable complement to the CA approach and can be used when the assumptions of parallel response curves are not met.

## 4 Conclusion

Overall, the GRA model developed as part of this research successfully predicts the effects of the mixtures, with the % of variance explained (RSQ-values) ranging from 84% to 99%. Furthermore, GRA outperforms the CA model applied by default (% variance explained 23%–98%) in describing the responses of non-equipotent mixtures of partial and complete agonists. The assumption that each component of the mixture can be considered as a dilution of the other is therefore not validated. This does not necessarily exclude the CA hypothesis, nor does it validate the IA ones. Recombinant cells used in the ERα-CALUX bioassay contain a stably transfected firefly luciferase reporter gene that responds to chemicals that can bind to the ER and activate it, resulting in the induction of luciferase gene expression. This induction occurs in a time-, concentration- and chemical-specific manner, which could lead to dissimilarities depending on the mixtures of agonists tested (Rogers and Denison, 2000; Besselink, 2015).

These results highlight the importance of applying an additive model for xenoestrogen risk assessment, but that care should be taken at low doses, as mixtures may exhibit joint behaviour if the chemicals are combined below their individual level with no observable adverse effects (NOAEL). Additionally, the compounds Triadimenol and BHT should be studied more intensely, as they show potential signs of synergy when tested in combination. Taken together, these results also underscore the need to broaden the approach to ED risk assessment, going beyond the treatment of individual compounds, for example, using adverse reaction pathways (AOPs) as tools to support cumulative risk assessment of co-exposure in food and food contact materials.

## Data Availability

The datasets presented in this study can be found in online repositories. The names of the repository/repositories and accession number(s) can be found in the article/Supplementary Material.
