# Supplementary material for: Prediction and assessment of xenoestrogens mixture effects using the in vitro ERα-CALUX assay
Source: Front Toxicol. 2023 Dec 8;5:1252847. doi: 10.3389/ftox.2023.1252847 (PMC10739317; doi:10.3389/ftox.2023.1252847)
Supplement: Supplementary file 1 [file DataSheet1.PDF]

Supplementary Material

Prediction and assessment of xenoestrogens mixture effects using the in vitro ERα-CALUX assay.

Marc Elskens[1], Imke Boonen[1], Steven Eisenreich[1,2]

[1]Laboratory for Analytical and Environmental Chemistry, Chemistry Department, Vrije Universiteit Brussel, Pleinlaan 2, B-1050 Brussels, Belgium

[2]Hydrology & Hydraulic Engineering Department, Vrije Universiteit Brussel, Pleinlaan 2, B-1050 Brussels, Belgium

1 Selection of food samples and food contact materials

For the selection of food matrices, an extensive literature search was conducted to determine food matrices likely to contain phytoestrogens, biocides and plant protection products. Relevant food samples were furthermore selected based on the Second French Total Diet Study and the European Union reports on pesticide residues in food from 2015, 2017 and 2018. Next, according to the latest Nation Food Consumption Survey, the highly consumed food was selected. Since each food item can be sold under various brands, the best-selling products were identified using the Euromonitor database. House brands were also selected to be as representative as possible. The sampling took place in the major supermarkets in Brussels, which cover 84% of the market share.

2 Exposure assessment and risk characterization

The exposure evaluation was realized using national representative food consumption data of the second Belgian food consumption survey (2014) for persons aged between 3 and 65 years [Bel et al., 2016].

3 References

S. Bel, S. Van den Abeele, T. Lebacq, C. Ost, L. Brocatus, C. Sti´evenart, E. Teppers, J. Tafforeau, and K. Cuypers. Protocol of the belgian food consumption survey 2014: objectives, design and methods. Archives of Public Health, 74(1):1–11, 2016.

4 Supplementary Tables

Table 1. Concentration response curve parameters for selected chemicals showing agonistic activity on ERα-CALUX. The concentration response curves were measured in triplicate. Values are mean (95% CI). *NA* = no activity detected.

Chemicals	Efficacy	Potency [nM]	Slope	RMSE[1]	RSQ[2]
17β-estradiol	0.98 (0.6)	0.013 (0.002)	1.6 (0.2)	1.6-3.1	0.992–0.999
Daidzein	1.98 (0.4)	1976 (570)	1.3 (0.4)	3.2-4.5	0.990-0.998
Genistein	2.14 (0.5)	530 (100)	0.5 (0.2)	2.7-6.9	0.990-0.996
Equol	0.97 (0.1)	502 (70)	1.6 (0.3)	1.0-1.5	0.992-0.999
6-PN	0.87 (0.2)	147 (52)	1.4 (0.3)	2.8-6.9	0.990-0.998
8-PN	0.77 (0.1)	13 (4)	1.3 (0.2)	2.8-6.9	0.996-0.999
Kaempferol	0.64 (0.2)	5899 (640)	5.2 (0.9)	2.5-6.7	0.989-0.995
Enterolactone	1.0 (fixed at bond)	>5E5 (fixed at bond)	0.9 (0.2)	1.5-3.5	0.957-0.998
Benzophenone	0.92 (0.2)	30775 (4459)	1.2 (0.2)	1.3-4.7	0.968-0.980
DEHP	0.02	3000000	1.2	1.3	0.968
DIBP	1.0 (fixed at bond)	12809262 (779285)	1.1 (0.1)	1.8-3.2	0.968-0.997
DBP	1.0 (fixed at bond)	11700717 (497239)	1.2 (0.3)	1.7-3.2	0.968-998
BBP	1.0 (fixed at bond)	31659160 (408585)	1.1 (0.2)	3.5-4.7	0.970-0.981
BHT	1.0 (fixed at bond)	>5E5 (fixed at bond)	1.6 (0.6)	1.7-3.8	0.937-0.988
Triadimenol	1.0 (fixed at bond)	100639 (14487)	3.9 (0.9)	0.5-1.5	0.996-0.998

(1)RMSE indicates the absolute fit of the model to the data, i.e. how close the observed data points are to the values predicted by the model. As the square root of a variance, the RMSE is the standard deviation of the unexplained variance and a robust measure of the accuracy with which the model predicts the response. (2)RSQ indicates the percentage of the variance explained by the logistic regression model.

Table 2. Concentration response-curves generated by CA and GRA for equipotent binary mixtures of fictitious agonists/antagonists. Input represents the individual parameter values introduced into the models. Output represents the actual parameter-values deduced from the mixing curve. DF stands for degree of freedom. Estimated parameters are best fitted values ± standard error.

Model	Input	Output	Estimated parameters			Goodness-of fit parameters		
	$(c_i, \ a_i, \ n_i)$	$(c, \ a, \ n)$	c	a	n	RMSE	RSQ	DF
CA	$\begin{pmatrix} 0.1 \\ 1 \end{pmatrix}$	$(0.09, \ 1, \ 1)$	0.09±0.01	1.02±0.02	1.0±0.1	3.033	0.9972	6
GRA#1	$\begin{pmatrix} 0.1 & 1 & 1 \\ 1 & 1 & 1 \end{pmatrix}$	$(0.09, \ 1, \ 1)$	0.08±0.01	0.98±0.02	1.0±0.1	2.857	0.9974	6
GRA#2	$\begin{pmatrix} 0.1 & 0.5 & 1 \\ 1 & 0.5 & 1 \end{pmatrix}$	$(0.06, \ 1, \ 0.5)$	0.07±0.01	1.03±0.03	0.7±0.1	3.950	0.9956	6
GRA#3	$\begin{pmatrix} 0.1 & 2 & 1 \\ 1 & 2 & 1 \end{pmatrix}$	$(0.09, \ 1, \ 2)$	0.10±0.01	1.03±0.03	2.5±0.6	5.300	0.9921	6
GRA#4	$\begin{pmatrix} 0.1 & 1 & 0.5 \\ 1 & 1 & 0.5 \end{pmatrix}$	$(0.09, \ 1, \ 2)$	0.11±0.03	0.52±0.03	0.9±0.02	0.410	0.9998	6
GRA#5	$\begin{pmatrix} 0.1 & 1 & 2 \\ 1 & 1 & 2 \end{pmatrix}$	$(0.09, \ 1, \ 2)$	0.10±0.01	2.1±0.03	1.1±0.08	4.628	0.9984	6
GRA#6	EC_{50} $(1 \ \ 0 \ \ 1)$	$(2, \ 1, \ 1)$	2.07±0.23	0.0±0.05	0.9±0.1	2.747	0.9958	6
GRA#7	EC_{50} $(1 \ \ 1 \ \ 0.5)$	$(4, \ 1, \ 0.5)$	3.42±1.52	0.0±0.05	0.5±0.1	4.889	0.9903	6
GRA#8	EC_{50} $(1 \ \ 0 \ \ 2)$	$(1.2, \ 1, \ 4)$	1.15±0.06	0.0±0.05	3.7±0.5	3.859	0.9947	6
GRA#9	$(0.1 \ \ 1 \ \ 1)$ EC_{10}	$(0.1, \ 1, \ 1)$	0.09±0.01	0.99±0.01	0.91±0.10	2.815	0.9975	6
GRA#10	$(0.1 \ \ 1 \ \ 1)$ EC_{50}	$(0.2 \ \ 1, \ 1)$	0.29±0.04	1.08±0.26	0.9±0.09	3.291	0.9968	6
GRA#11	$(0.1 \ \ 1 \ \ 1)$ EC_{90}	$(1, \ 1, \ 1)$	1.13±0.17	1.02±0.39	0.9±0.14	4.041	0.9937	6

Table 3. Contents and concentrations of mixtures based on the concentration ratios found by chemical analysis in food samples. * E2 is maintained at its EC_{50}

Mix	Compounds	%	Conc. In Food [mg/kg]	Food	Con. In Mix [nM]	Mod	RMSE	RSQ
#a	Daidzein<br>Equol<br>8-PN<br>Kaempferol	14<br>5.5<br>0.1<br>80	-	-	331<br>127<br>2<br>1858	GRA<br>CA	3.3<br>6.4	0.9984<br>0.9291
#b	Daidzein<br>Equol<br>8-PN<br>Kaempferol	22<br>6.1<br>0.16<br>72	-	-	1794<br>502<br>13<br>5899	GRA<br>CA	7.9<br>4.0	0.9667<br>0.9872
#c	Daidzein<br>Equol<br>8-PN<br>Kaempferol<br>E2*	22<br>6.1<br>0.16<br>72<br>-	-	-	7176<br>2008<br>52<br>23596<br>0.013	GRA<br>CA	4.8<br>5.0	0.9819<br>0.9624
#1	Daidzein<br>Genistein	41<br>59	44<br>62	Greek yoghurt	56<br>91	GRA<br>CA	2.8<br>10.3	0.9247<br>0.5553
#2	Daidzein<br>Genistein	38<br>62	2.2<br>3.6	Vegan minced meat	56<br>92	GRA<br>CA	2.9<br>9.0	0.9372<br>0.6278
#3	Daidzein<br>Genistein	57<br>43	0.0065<br>0.005	Princess beans	83<br>64	GRA<br>CA	2.9<br>8.7	0.8465<br>0.4403
#4	Daidzein<br>Genistein<br>BEHP	35<br>27<br>38	0.0065<br>0.005<br>0.007	Princess beans	3565<br>2743<br>3840	GRA<br>CA	6.5<br>61.8	0.9862<br>0.5137
#5	Daidzein<br>Genistein<br>6-PN<br>Kaempferol	41<br>58<br>0.12<br>0.02	44<br>62<br>0.13<br>0.02	Greek yoghurt	1660<br>2341<br>5<br>0.75	GRA<br>CA	7.6<br>20.6	0.9928<br>0.9687
#6	Daidzein<br>Genistein<br>6-PN<br>Enterolactone	37<br>61<br>1.2<br>0.5	2.2<br>3.7<br>0.07<br>0.03	Vegan minced meat	2855<br>4653<br>89<br>38	GRA<br>CA	12.6<br>36.7	0.9666<br>0.9515
#7	DEHP<br>DIBP<br>DBP<br>BBP	49<br>16<br>28<br>6	0.33<br>0.11<br>0.19<br>0.04	Peanut oil	19699<br>6556<br>11429<br>2346	GRA<br>CA	1.7<br>14.7	0.9925<br>0.4971
#8	DEHP<br>DIBP<br>DBP<br>Benzophenone	43<br>15<br>25<br>17	0.33<br>0.11<br>0.19<br>0.13	Peanut oil	17485<br>5828<br>10159<br>6844	GRA<br>CA	3.5<br>20.5	0.9393<br>0.3579
#9	Daidzein<br>Genistein<br>DEHP<br>DIBP<br>DBP	41<br>58<br>0.03<br>0.03<br>0.02	44<br>62<br>0.029<br>0.031<br>0.025	Greek yoghurt	1250<br>1762<br>0.82<br>0.88<br>0.70	GRA<br>CA	13.1<br>66.8	0.9831<br>0.5140
#10	Daidzein<br>Genistein<br>6-PN<br>Enterolactone<br>BHT	25<br>40<br>0.7<br>0.3<br>34	2.2<br>3.7<br>0.07<br>0.03<br>3.1	Vegan minced meat	2279<br>3713<br>71<br>31<br>3103	GRA<br>CA	23.9<br>52.8	0.9431<br>0.6355
#11	Daidzein<br>Genistein<br>DIBP<br>Benzophenone<br>Triadimenol	17<br>7<br>24<br>21<br>31	0.005<br>0.002<br>0.007<br>0.006<br>0.009	Choco-late mousse	13077<br>5231<br>18308<br>15692<br>23539	GRA<br>CA	23.3<br>54.8	0.9663<br>0.6079

#12	DEHP<br>DIBP<br>DBP<br>BHT<br>Benzophenone	49<br>10<br>22<br>14<br>5	0.049<br>0.096<br>0.023<br>0.014<br>0.005	Choco-<br>late ice<br>cream	61215<br>11776<br>27602<br>17420<br>6624	GRA<br>CA	5.3<br>34.8	0.9419<br>0.2322
